# A Comprehensive Review of the Genetic Etiology and Management of Orofacial Clefts

**DOI:** 10.1002/pdi3.70037

**Published:** 2026-04-16

**Authors:** Emily Kim, Rebeka Dejenie, Caroline Kim, Russell Reid

**Affiliations:** ^1^ The University of Chicago Medical Center Chicago Illinois USA; ^2^ Independent Researcher; ^3^ Laboratory of Craniofacial Biology and Development, Section of Plastic and Reconstructive Surgery, Department of Surgery The University of Chicago Medical Center Chicago Illinois USA; ^4^ Section of Plastic and Reconstructive Surgery, Department of Surgery Chicago Illinois USA

**Keywords:** cleft lip, cleft palate, orofacial clefts

## Abstract

Cleft lip (CL) and cleft palate (CP), collectively referred to as orofacial clefts (OFCs), are among the most common birth defects and can have significant effects on speech, nutrition, and physical and psychosocial development. Manifestation, classification, and treatment plans of OFCs are diverse and not standardized. Recent technological advancements and genetic testing have enabled researchers to propose relationships between certain genes and the development of OFCs. Potential genetic precursors for OFCs have been found to be numerous and diverse. Treatment strategies are also numerous and vary greatly between individuals. In this review article, we summarize the most commonly discussed genetic factors in the literature, variation in classification, and advances in management.

## 1 Introduction and Epidemiology

Orofacial cleft (OFC) is a disorder that comprises various abnormal developments of the upper lip and/or hard and soft palate. Cleft lip (CL) occurs when the tissue of the lip fails to fuse during development, resulting in the division of the upper lip [1]. Clefting of the upper lip results in the deformation of nostrils and the nasal sill. In extremely rare cases, it can occur in the lower lip. CL extends from the lip through the nasal floor and is considered incomplete if the cleft does not extend through the nasal floor [2]. Cleft palate (CP) occurs when the lateral palatine shelves do not fuse, incomplete clefting of the hard and/or soft palate.

OFCs occur in roughly 0.60 births per thousand with a range from 0.13 to 1 per 1000 births globally across regions [3, 4]. CL currently affects 600–800 out of 1000 births, whereas cleft palate affects 1 in 2000. Of all OFC cases, 15% are CP only, 45% are both CL and CP, and 40% are CL only [5]. There are many classification systems for OFCs. Syndromic OFCs refer to cases where OFCs constitute one clinical finding among a constellation of findings and are thought to have a genetic cause. Nonsyndromic OFCs refer to cases where the OFC is an isolated finding. Understanding the development and treatment of OFCs is paramount as OFCs have lasting effects on the patient's facial growth, function (airway, auditory, mastication, and deglutition, as well as speech development) and psychosocial wellness. It follows that this condition mandates high‐level multidisciplinary care and good patient caregivers, and multiple specialists, parents/guardians, and multiple specialists.

OFCs have been found to correlate with biological ancestry. In the United States, Native Americans and Alaskan Natives have the highest incidence with roughly 1.2 per 1000 births [6]. The incidence for Caucasians is slightly lower at 1 per 1000 live births or as low as 0.8 per 1000 [6, 7, 8]. Incidence for African Americans tends to be relatively low with one study reporting 0.54 per 1000 and another study reporting 0.66 per 1000 births [6, 7, 9]. Hispanic populations have a incidence around 0.75 to 1.1 per 1000 births. Asian and Pacific Islander populations in the US have a reported prevalence between 0.47 and 0.996 per 1000 births [6, 10]. Two of the most recent compilations of OFC prevalence data in the US both used the National Birth Defect Prevention Network Data and categorized the cases by diagnosis codes, but one examined a 12‐year span and another a 4‐year span, which may be the cause in the discrepancies in prevalence estimates. The occurrence of OFC also correlates with sex. CL is almost twice as prevalent in males than females, whereas CP is more prevalent in females than males, potentially due to the differing rates of palate development [11, 12, 13, 14]. Unilateral cleft is the most common overall and tends to be on the left side more frequently, making up 66% of unilateral cases of CL/P [11]. OFCs are also more common in rural populations and among individuals with lower socioeconomic status [9, 15, 16, 17]. Lower socioeconomic status has been associated with increased likelihood of a lack of adequate nutrition and vitamins as well as increased environmental risk factors during fetal development which can cause OFCs [18].

## 2 Embryology

Lip development occurs from the fourth through eighth week of fetal development. It begins with the development of the five facial primordia: the frontonasal prominence, the bilateral maxillary prominences, and the bilateral mandibular prominences. The frontonasal prominence forms the medial and lateral nasal prominences that will become the forehead, nose, and top of the mouth. The maxillary prominences will form the cheeks, and the mandibular prominences will become the lower portion of the face and jaw. At the end of the sixth week to the beginning of the seventh week, the maxillary prominences grow and fuse with the medial nasal prominence. In the eighth week, the maxillary prominences grow further and fuse with the lateral nasal prominences [19]. CL occurs when the maxillary prominence fails to fuse with the nasal prominences [20].

**FIGURE 1:**
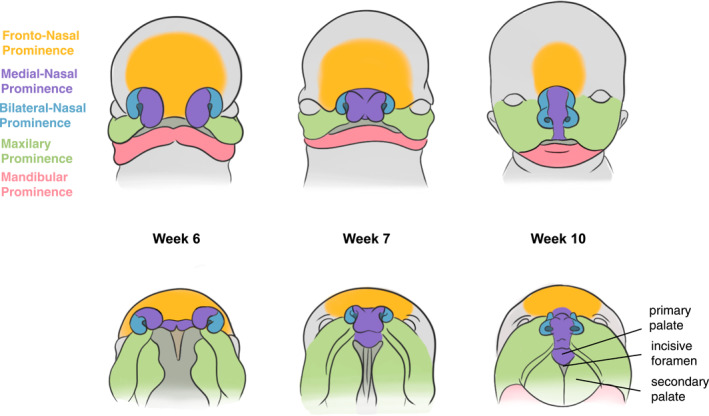
Fetal development of the face and palate.

Palate development occurs approximately between the fifth and twelfth weeks of embryonic development. The key steps of development occur during weeks 6 through 10, as seen in Figure 1. The fusion of the maxillary prominences with the medial nasal prominence forms a mass of mesenchymal tissue that eventually forms the primary palate. Part of the maxillary prominence also becomes the secondary palate. These projections start on either side of the developing mouth and will rotate and elevate to the correct position and height. Thinning of the tissue via cellular apoptosis and regeneration allows the tissue to fuse in the midline of the mouth. Fusion begins in the ninth week and concludes in the 12th week [19]. The anterior portion of the palate will ossify into the hard palate whereas the posterior third will become the soft palate and uvula. CP occurs when these fusion processes fail [20]. The timing of the improper fusion determines the type, degree, and location of the CP [21].

## 3 Etiology

### 3.1 Genetic Factors

Palate fusion is a complex process that depends on specific cells and signaling pathways. Medial edge epithelial (MEE) cells are found where the two sides of the palate meet. These cells are crucial for fusion. Over time, they disappear from the seam through cell migration, epithelial‐mesenchymal transition (EMT), and/or programmed cell death. The transforming growth factor (TGF)‐β pathway stimulates various signaling networks to control cell fate determination, growth, and differentiation. Signaling by TGF‐β plays an important role in the palatogenesis [22].

TGF‐β epithelial signaling that guides myogenesis in the soft palate is controlled by Wnt/β‐catenin signaling, playing an essential role in palatal development mediating [23]. β‐catenin and several Wnt ligands and receptors are expressed in the MEE cells, and epithelial‐specific inactivation of β‐catenin results in CP formation, indicating that canonical Wnt/β‐catenin signaling is a critical regulator of palate fusion through its role in maintaining TGF‐β3 expression in MEE [24].

Although syndromic cases are more explicitly linked to a specific genetic condition, nonsyndromic cases also have a genetic component. Some of these genetic components are related to the TGF and Wnt pathways. There is a rough positive correlation between the proportion of relatives with OFC and the odds of any offspring also having OFCs. The discovery of numerous candidate genes associated with OFC has led to a better understanding of the molecular mechanism involved in the normal craniofacial development and OFC formation. Some of the most common genes associated with OFCs are listed in the subsequent paragraphs. Additional genes with potential correlation have been listed in Table 1.

**TABLE 1:** Overview of various genes potentially correlated with the development of orofacial clefts.

Gene	Location	Function	Pathogenic variants
*IRF6*	1q32	Palate fusion, broadly a transcription activator in skin tissue	Multiple: Naicker et al. [25], Park et al. [26], Rahimov et al. [27], Wattanawong et al. [28], and Zhang et al. [29]
*8q24*	8q24	Noncoding region (no function so far)	rs987525: Rojas‐Martinez et al. [30], Salagovic et al. [31], Souza et al. [32], and Wattanawong et al. [28]
*TGFA*	2p13.3	Transgenic growth factor alpha, participates in palette fusion	Multiple: Jugessur et al. [33], Kim et al. [34], and Carter et al. [35]
*TGFB1*	19q13.2	Cell proliferation, differentiation, platelets, and immune system function	Multiple: Fonseca‐Souza et al. [36] and Stoll et al. [37]
*TGFB3* [38]	14q24.3	Cytokine for cell differentiation and embryogenesis	Multiple: Jugessur et al. [33] and Ichikawa et al. [39]
*SOX9* [40, 41]	17q24	Transcription factor for cell differentiation such as cranial neural crest cells	n/a
*SUMO1* [42]	2q33.1	Transcriptional regulation, apoptosis, protein stability, and nuclear transport	Multiple: Carter et al. [35], Song et al. [42], and Tang et al. [43]
*MSX1*	4p16.2	Transcription factor used in palate fusion	Multiple: Jagomägi et al. [44], Jugessur et al. [33], Kim et al. [45], and Suazo et al. [46]
*PAX7* [47]	1p36.13	Transcription factor expressed in neural crest tissues [48]	Multiple: Böhmer et al. [49], Khan et al. [50], and Sull et al. [47]
*BMP4*	14q22.2	Bone morphogenetic protein 4	Multiple: Hao et al. [51], Kempa et al. [52], Suazo et al. [53], and Wang et al. [54]
*NOG* [55]	17q22	Noggin protein, involved in nerve, muscle, and bone tissue	Multiple: Leslie et al. [56], Song et al. [42] and Tang et al. [43]
*VAX1* [57]	10q25	Ventral anterior homeobox	rs7078160: Nassar et al. [58], Peng et al. [59], Sabbagh et al. [60], Wang et al. [61], and Zhang et al. [57]
*MTHF*	1p36	Folate and homocysteine metabolism [62]	rs1801133 and rs1801131: Amooee et al. [63], de Aguiar et al. [64], Niktabar et al. [65], Jagomägi et al. [44], Li et al. [66], and Rafik et al. [67]
*RARA* [68]	17q21.2	Retinoic acid receptor alpha	n/a
*WNT9B*	17q21.3	Neural differentiation through retinoic acid regulation	Multiple: Jain et al. [70], Menezes et al. [71]
*ABCA4* [72]	1p22.1‐31	ATP‐binding cassette subfamily A, member 4 which has interactions with *IRF6* and folic acid metabolism [73]	rs560426: Fontoura et al. [74], Beaty et al. [75], Wu‐Chou et al. [76], and Zawiślak et al. [77]
*CRISPLD2* [78]	16q24	Cysteine‐rich proteins with interactions in folic acid pathway [79]	Multiple: Assis Machado et al. [80], Mijiti et al. [81], Neela et al. [82], Shen et al. [83], and Letra et al. [84]

*Interferon regulatory factor 6* (*IRF6*), a transcription factor localized in 1q32.4‐41, which codes for transcription factors, is one of the most commonly cited genes associated with syndromic and nonsyndromic OFCs [85, 86, 87]. It plays a role in the fusion of the medial palatal shelves by involving in TFG‐β3 mediated palate fusion [88]. It was first found to be associated with syndromic OFC in Van Der Woude syndrome; an singular nucleotide polymorphism (SNP) associated with this gene is responsible for about 2% of syndromic cases [85, 87, 88]. There are 4 SNPs that have significant impact on the development of OFCs [75]. OFCs caused by *IRF6* mutations are more prevalent in Asian populations [11].

There is some evidence, although disputed, that 8q24 interacts with *IRF6* to cause cleft lip with or without cleft palate CL/P [89, 90]. 8q24 is a gene desert, which does not encode for any genes. It has been linked with CL/P, mostly in populations of European ancestry [30, 91, 92, 93].

Many genes associated with fetal development have been found to be associated with CL/P. The *transforming growth factor alpha* (*TGFA*) on 2p13.3 is responsible for platelet development and fusion; it has been found to be associated with CL/P [94, 95]. Maternal smoking during fetal development has been found to have a correlation with altered *TGFA* sequence and thus on CL/P development [96, 97, 98].

Another gene importance for fetal development is muscle segment homeobox (MSX)‐1, one of the MSH homeobox genes located on 4p16.1, which is responsible for cell differentiation and phase separation, essential for palate fusion [99]. It activates cyclin D1 which inhibits cell differentiation. In a murine study, *MSX1* has been found in the anterior palatal mesenchyme and depends on BMP signaling [100]. In a study of Dutch families, a nonsense mutation in *MSX1* can result in CL/P or cleft palate only (CPO) [101, 102]. Several other studies have found a correlation between the *MSX1* gene and CL/P in other populations [103, 104, 105].

*BMP4* on 14q22.2 is a transforming growth factor beta and is responsible for bone development specifically cartilage and tooth development and fracture repair. BMP signaling also helps control the development of the maxillary and mandibular processes which are crucial for palate and lip development [106, 107]. Several studies have found linkages to *BMP4* mutation and CL/P [108, 109, 110]. There is also some evidence that rs17563, one of the SNPs linked to *BMP4*, is associated with CL/P [51, 111]. In a murine model, *BMP4* and *MSX* played a significant role in palate development, further supporting the conclusions of the population studies [112].

*SUMO1* located on 2q33.1 facilitates transcriptional regulation, apoptosis, protein stability, and nuclear transport by encoding small ubiquitin‐like modifier‐1 SUMO1 protein for sumoylation. The sumoylation of proteins was involved with palate development, such as MSX1 and SOX9 [113]. SUMO1 has had some success as an indicator for CL/P [35, 42]. Four different SNPs linked to SUMO1 were found to have an association with CL/P [43].

*MTHFR* on 1q36 encodes for an enzyme involved in folic metabolism. Folic acid levels have been noted to have an effect on CL/P development [114]. Some studies have reported associations between specific SNPs on *MTHFR*, such as rs180113, c.G586A, and p.G196S, that could lead to the development of CL/P [102, 111, 114, 115, 116, 117, 118]. OFCs correlated with *MTHFR* mutations are reported in Caucasian populations more frequently than those in Asian populations [63].

### 3.2 Environmental Factors

OFCs are multifactorial; therefore, there are several environmental factors during fetal development that contribute to the manifestation of OFCs. As mentioned, some environmental factors in combination with certain genetic mutations have been found to increase the likelihood of OFC development.

Smoking is associated with OFCs, with more association with CL/P than CPO [119, 120, 121]. Some studies suggest that the effects of smoking are exacerbated when *TGFA* mutations are present [33, 96, 122, 123]. There has also been some evidence that mutations in *TBK1* on chromosome 12 and in *ZNF36* on chromosome 18 in conjunction with smoking also increase the odds of OFC development [124, 125, 126]. For *MSX1*, the odds of OFC formation increased by a factor of 7.16 with maternal smoking [127].

Maternal consumption has also been attributed to causing OFCs, especially binge drinking [128, 129]. Specifically *MLLT3* and *SMC* on chromosome 9 are thought to elevate the risk of OFC under alcohol exposure during fetal development, though data are limited [126]. However, this may be inconclusive as several meta‐analysis studies have reported that there is no consistent correlation between maternal alcohol consumption and clefting [130, 131]. A meta‐analysis of 33 studies found an odds ratio (OR) of 1.00 (95% CI 0.86–1.16) for (CL/P), indicating no increased risk, and 1.05 (95% CI 0.92–1.21) for CPO, also suggesting minimal risk [130, 131]. Another meta‐analysis of nine studies have found similar results, with ORs of 1.00 (95% CI 0.87–1.15) for CL/P and 1.02 (95% CI 0.92–1.14) for CPO [130, 131]. However, some studies within this analysis suggested a potential increased CL/P risk with high alcohol consumption or binge drinking [130, 131].

Vitamin deficiency, particularly vitamin A or folic acid, has also been reported to contribute to OFCs, though the data are varying. Folic acid deficiency increases the risk of CL/P, although one case–control study found that periconceptional folic acid supplements had minimal impact withORs of 1.01 (95% CI 0.82–1.24) for CL/P and 1.02 (95% CI 0.77–1.34) for CPO, revealing no significant change in risk [97]. However, one study found higher dietary folate intake was protective, with an OR of 0.56 (95% CI 0.3–0.9) for CL/P, suggesting a reduced risk [114]. Additionally, higher intakes of dietary nutrients, such as iron and riboflavin, were associated with reduced CL/P risk, suggesting a role for broader nutritional status [97]. Polymorphisms in the *MTHFR* gene, such as C677T, impair folate metabolism and may play a role in increasing CL/P risk. One meta‐analysis analyzing the association between *MTHFR* polymorphisms and nonsyndromic CL/P in Asian children and mothers reported a 1.4‐fold increased risk (OR 1.41, 95% CI 1.23–1.61) in children, and 1.7‐fold increased risk (OR 1.70, 95% CI 1.19–2.42) in mothers [132]. However, the *MTHFR* A1298C polymorphism showed no association.

Certain drugs, such as antifolates, steroids, and retinoic acids, have been found to impact the development of OFCs [133, 134, 135, 136, 137, 138]. Antiepileptic drugs have also been found to be teratogens. Specifically, topiramate's effect on *TGFB1* and *SOX9* regulation and valproate's effect on Beta‐catenin signaling during cell differentiation have been linked to OFCs (11.2 in 1000 births) [40, 139, 140, 141]. One study has reported an OR of 5.4 for the risk of OFC due to topiramate use [142]. For valproate use, the corresponding OR for CP is 5.2 [143]. Additionally, maternal diabetes or obesity has been associated with OFCs [144, 145, 146, 147]. Other teratogenic drugs, such as selective serotonin reuptake inhibitors (SSRIs) or serotonin‐norepinephrine reuptake inhibitors (SNIRs), including fluoxetine (OR 1.34 and 95% CI 1.19–1.51), sertraline (OR 1.25 and 95% CI 1.16–1.34), and citalopram (OR 1.28 and 95% CI 1.11–1.47), have been associated with an increased risk of OFCs [148]. Previous studies have associated benzodiazepine use with CL/P as a meta‐analysis previously reported an OR of 1.79 (95% CI 1.13–2.82) for oral clefts in case‐control studies [149]. However, cohort studies, including those in an updated meta‐analysis involving over one million pregnancies, found no association. However, other studies have not been able to conclude a relationship between benzodiazepine use and CL/P [150, 151]. These findings underscore the need for comprehensive risk assessments and counseling for pregnant women regarding medication use.

## 4 Clinical Diagnosis and Classification

CL/P are ideally diagnosed prenatally and confirmed at birth by examination. Early diagnosis allows for adequate time for parental counseling and the development of a long‐term treatment plan [152]. 2D ultrasound is the most common method of tracking fetal development. Although CL/P can be detected by a 2D ultrasound as early as 16 weeks of gestation, diagnosis of CL/P with this method is dependent on the operator as well as conditions inside the uterus related to fetal position, amount of amniotic fluid, and type of cleft [153]. Additionally, 3D ultrasound has been found to significantly increase the rate of diagnosis of CL/P [154]. Fetal magnetic resonance imaging (MRI) is also used in adjunct to ultrasounds to increase the accuracy of diagnosis as they are less dependent on the conditions and positions of the baby *in utero* [155]. However, the use of MRI as a prenatal tool is limited by cost and accessibility.

OFC manifests as either unilateral or bilateral and as either complete or incomplete. A complete cleft involves the primary and secondary palate, typically manifesting as a notch in the skin from the lip that extends up into the nostril, whereas an incomplete cleft occurs only in secondary palate manifesting as a notch in the lip that does not extend to the nostril [156]. At birth, a CL will typically manifest itself with the forward or outward rotation of the premaxilla and the deformation of the upper lip and nose. The philtrum is typically shortened and the vermilion is imbalanced with the medial segment being relatively deficient compared to the lateral segment. In bilateral clefts, the cupid's bow is obliterated, the philtrum is diminutive, and the orbicularis oris muscle is severely disrupted. Additionally, the lateral portion of the lip will be fused to the alar bases instead of being continuous with the medial segment of the palate. Occasionally, a “Simonart's band” (a soft tissue bridge of any size spanning the nasal sill) may be present in either unilateral or bilateral cases [157].

Other features of CL include multiple abnormalities in the nasal area. Often, CL deformities include nasal findings such as deficiency and descent of the lower lateral cartilage on the affected side, asymmetry of the nasal tip and nostrils, malpositioning of the alar base with increased base width, shortened columella on the cleft side, and caudal septal deviation toward the noncleft side.

There is no one standardized system of classifying CL/P. Some more commonly used classification systems include Veau classification, Kernahan and Stark classifications, and the International Statistical Classifications of Diseases and Related Health Problems (ICD‐10) [158].

**FIGURE 2:**
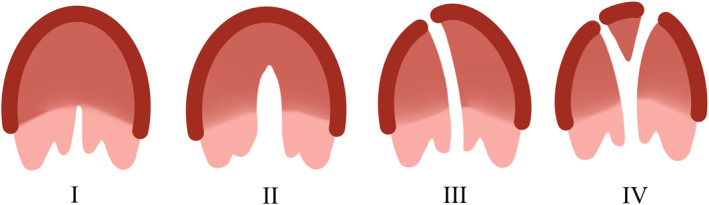
The Veau classification system.

In the Veau classification, there are four different kinds of CP as shown in Figure 2. Class I is an incomplete cleft where only the soft palate is compromised. Class II is the clefting of the secondary palate both hard and soft. Class III is unilateral clefting of the primary and secondary palate, and Class IV is bilateral clefting of the primary and secondary palate [159].

**FIGURE 3:**
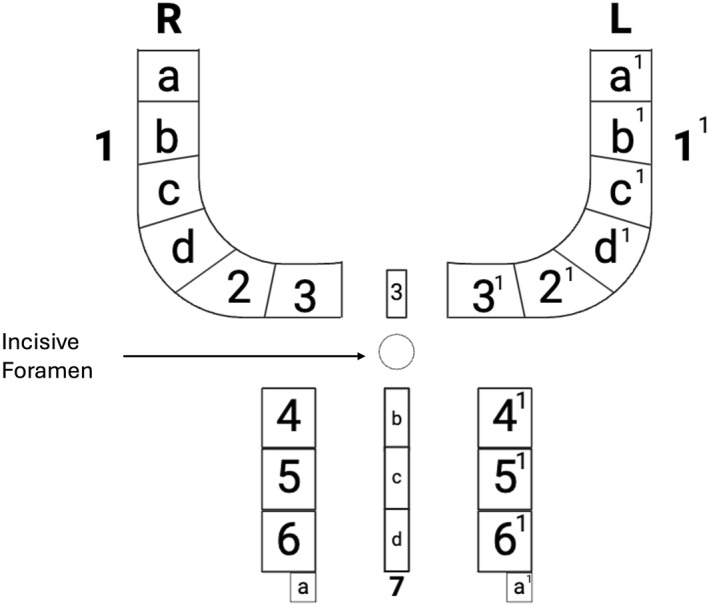
The Smith modified Kernahan's Y classification. (a–d) (1) complete cleft; (2) alveolus; (3) primary palate; (4) cleft up to the palatine process of the maxillary bone; (5) cleft up the palatine process of the palatine bone; and (6–7) soft palate cleft, a submucous cleft.

Kernahan and Stark classification is based on embryologic development [160]. It has been modified a number of times, by Kernahan et al. in 1971 himself and later Smith et al. in the work of Karataş et al. in 1998 and in the work of Khan et al. in 2013 [161, 162]. As shown in Figure 3, the Smith modified system describes the location of the CP using several numbers and letters to differentiate the right and left, soft and hard, and depth of cleft.

ICD‐10 is a registration process for coding and not technically a classification system. It lacks the specificity that other systems above possess, but it is often used out of necessity as mandated for insurance reimbursements [158].

## 5 Management

Treatment of cleft lip and palate is a multidisciplinary effort. It may not only require multistaged surgeries, but the coordination and intervention of many specialties including plastic surgery, genetics, speech and feeding therapy, orthodontics and oral surgery, audiology, ENT (ear, nose, and throat), psychiatry, and social work as well as ongoing monitoring by parents/guardians to obtain the optimal cosmetic and functional outcomes.

In general, CL repair occurs first, within the first 3–6 months of age. Later, around 6–12 months of age, surgical CP repair is performed depending on cleft morphology and the patient's comorbidities. Simultaneous surgeries to prevent ear infections performed by pediatric otolaryngologists (i.e., myringotomy and pressure equalization tube placement) are common in patients with OFCs and critical to prevent hearing loss and enhance speech and language development in these patients. Dental and orthodontic interventions are vital in terms of maintaining tooth health, maxillomandibular arch coordination, and good occlusion. Esthetic reconstructive surgery, such as orthognathic surgery and cleft rhinoplasty, are later surgical interventions that can improve quality of life [163].

### 5.1 CL Repair

Recent preoperative developments have improved the functional and esthetic outcome of repaired CLs. Specifically lip taping, in place of presurgical infant orthopedics, has been adopted due to its simplicity and cost‐effectiveness [164]. Presurgical infant orthopedics utilizes nasoalveolar molding to reduce the size of the alveolar segment gap [165]. Lip taping aims to reduce the alveolar gap by stretching the facial skin with tape. The tape is initially applied from cheek to cheek and then primarily on the lips as the alveolar gap narrows [166].

Several CL repair techniques and modifications have been reported over the years, none of which have been rigorously supported by prospective data to be superior over the other, but rather depend on the skill of the surgeon performing the technique. For unilateral CL repair, the more recently described Fisher technique is used to improve symmetry between noncleft and cleft sides of the lip utilizing anatomical subunits of the lip and nose [167]. The Millard rotation advancement technique and its modifications are the most widely used repair approaches. Among these two popular techniques, the Fisher repair has demonstrated decreased rates of scar contracture and hypertrophy as well as a better esthetic outcome based on the Unilateral Cleft Lip Severity Index [168, 169, 170].

More recently, adjunct interventions, such as botulinum toxin into the orbicularis oris at the time of CL surgery, have been shown in a prospective study to improve scar outcomes theoretically by reducing muscle contraction and surgical wound tension [171].

Patient‐reported outcomes, such as CLEFT‐Q and Internal Consortium for Health Outcomes Measurement, are increasingly utilized to standardize and appraise to outcome of invasive and noninvasive interventions for CL [172, 173].

### 5.2 CP Repair

The timing of CP repair has been historically guided by Wilhelmsen and Musgrave's rule of tens, for example, the child should meet the following qualifications: weight ≥ 10 lbs (4.5 kg), hemoglobin at > 10 g/dL, and white blood cell count < 10,000 mm^3^ [174]. Currently, as long as the child demonstrates significant weight gain, and does not have comorbidities to preclude surgery, CP repair is performed in the 6–12 months age window, depending on the cleft morphology. To optimize speech development, surgery is optimally performed within 3–6 months of age, but at least before 12 months of age, to ensure proper speech development [175]. Prioritizing maxillofacial development tends to suggest surgery much later, after 12 months of age. Surgeries performed around the ages of 12–15 months tend to lead to optimal facial growth, but 86% of the cases examined had speech deficiencies resulting from glottal and pharyngeal issues [176]. In an attempt to optimize facial growth and speech development, some recommend staged surgeries performed in two steps. The first staged surgery occurs before 12 months to repair the soft palate, and sometimes ear tubes as an adjunct procedure to aid in Eustachian tube function, whereas the second staged procedure to close the hard palate occurs around 5–12 months of age [163, 177]. However, the overwhelming majority of cleft surgeons will complete total CP reconstruction by 12–15 months of age. The concept of timing and staging of CP repair remains an area of controversy and it can be multiple repairs.

The timing of CP repair influences OFC outcomes, with debate over single‐stage versus staged approaches. Single‐stage repair, typically at 6–12 months, aims to enhance speech and velopharyngeal function early, whereas staged approaches, separating soft and hard palate closure, may minimize maxillofacial growth restriction [178]. Recent cohort studies reporting single‐stage and two‐stage CP repairs showed no significant differences in speech or hearing outcomes, supporting surgeon and family preference in determining the timing of repair [178]. In terms of CP repairs, various techniques have been described, including a two‐flap palatoplasty technique with intralveolar veloplasty (Bardach), bipedicled flaps (von Langenbeck), and double‐opposing Z‐palatoplasty (Furlow) [179]. In addition to the traditional technique for CP repair, a buccal fat pad flap and a buccal myomucosal flap have been utilized to augment primary and secondary palatoplasty [180]. The most optimal technique is debated and can vary between patients. More long‐term data are needed to determine which technique is most optimal.

New techniques in OFC management are enhancing surgical outcomes by addressing long‐standing challenges in craniomaxillofacial reconstruction. 3D‐printed scaffolds enhance cleft lip and palate repair by providing patient‐specific models allowing surgeons to practice personalized medicine [181]. Also, 3D‐printed scaffolds may support alveolar cleft reconstruction and offer an alternative to autologous bone grafting [181, 182]. Botulinum toxin A (BoNT‐A) injections have also been explored as a way to mitigate cheiloplasty scars in patients with cleft lip and palate. One double‐blind randomized controlled study found that the administration of BoNT‐A resulted in narrower scars and improved cosmetic outcomes in patients with unilateral CL undergoing primary cheiloplasties [171].

Regardless of the technique, the basic principles of CP repair are: (1) Layer‐by‐layer watertight closure to minimize fistula occurrence, (2) velar muscle alignment and reconstruction, and (3) sufficient lengthening of the palate. Ultimately, these goals aim to support proper speech and velopharyngeal function.

After cleft lip and palate repairs are complete, the child enters the “functional years” in which speech therapy, continued audiology assessment, and orthodontic care are paramount. Some children may need secondary surgery for velopharyngeal insufficiency or hypernasal speech, such as pharyngeal flap reconstruction or sphincter pharyngoplasty. Orthodontic evaluation and intervention may consist of palatal expansion for transverse maxillary deficiency (Phase I), followed most often by secondary alveolar bone grafting, enhancement of arch coordination, canine substitution for lateral incisor agenesis and braces (Phase II), and then possibly Le Fort I surgical correction at skeletal maturity [183]. Alveolar bone grafting, commonly with iliac corticocancellous bone graft, supports permanent tooth eruption, maxillary stability, and further tooth movements. In the adolescent years, patients may need orthognathic surgery, midface distraction surgery, and/or cleft rhinoplasty surgery once skeletally mature [184]. Ongoing psychosocial rehabilitation for appearance‐related concerns and societal integration, often overlooked in the management of these patients with OFCs, are critical for the ultimate success of surgical outcomes, for the patient's self‐esteem, and productivity in society [185].

## 6 Conclusion

OFC is a functional and structural defect that profoundly impacts the physical and mental well‐being of affected individuals. Timely diagnosis of OFCs is paramount for effective treatment planning, which often necessitates interdisciplinary and sometimes multiprocedural approaches. Genetic association can give insight into the etiology of OFC development, aiding in early detection and intervention. Technological advancement has facilitated the identification of numerous genetic and environmental factors that contribute to OFC development. The most reliable early diagnostic techniques are 3D ultrasound or fetal MRI. Surgical treatment to restore anatomical integrity and function can vary depending on the individual and whether speech or facial development is prioritized, but it generally occurs between 6 and 15 months of age. The surgical technique chosen for closure depends on the patient, and more studies are necessary to ascertain optimal approaches. Additionally, comprehensive dental care plays a pivotal role in maintaining oral health. Jaw alignment and arch coordination are essential and can be treated nonsurgically or surgically. Ultimately, successful management of OFCs should restore functionality whereas concurrently considering facial aesthetics to improve physical development as well as social integration and mental well‐being. Future research should leverage CRISPR‐based gene editing to target specific genetic mutations associated with OFCs, potentially preventing their occurrence [186]. Multiomics approaches, integrating genomics, transcriptomics, and proteomics, could elucidate complex etiological pathways, enabling personalized interventions [187, 188]. Additionally, advancements in 3D bioprinting may offer novel reconstructive solutions, improving surgical outcomes [189]. These innovative strategies hold promise for enhancing both functional restoration and psychosocial well‐being for individuals with OFCs.

## Author Contributions

**Emily Kim:** writer, figures. **Rebeka Dejenie:** figures, editing. **Caroline Kim:** writer. **Russell Reid:** conceptualization of article, writer, editor.

## Funding

The authors have nothing to report.

## Ethics Statement

The authors have nothing to report.

## Conflicts of Interest

The authors declare no conflicts of interest.

## Data Availability

Data sharing is not applicable to this article as no datasets were generated or analyzed during the current study.
